# Generalization for both diurnal and nocturnal pollination in the mass‐flowering desert geophyte *Nerine laticoma* (Amaryllidaceae)

**DOI:** 10.1111/plb.70153

**Published:** 2025-12-12

**Authors:** G. L. Theron, C. Barker, M. Castañeda‐Zárate, C. Diller, S. Geerts, S. G. T. Klumpers, S. D. Johnson

**Affiliations:** ^1^ Agricultural Research Council Plant Health and Protection Pretoria South Africa; ^2^ Centre for Functional Biodiversity, School of Life Sciences University of KwaZulu–Natal Scottsville South Africa; ^3^ Department of Conservation and Marine Sciences Cape Peninsula University of Technology Cape Town South Africa; ^4^ Department of Botany and Zoology Stellenbosch University Matieland South Africa; ^5^ Department of Plant Protection Biology Swedish University of Agricultural Sciences Lomma Sweden; ^6^ Naturalis Biodiversity Center Leiden the Netherlands; ^7^ Present address: Department of Terrestrial Invertebrates National Museum Bloemfontein South Africa; ^8^ Present address: Department of Natural Sciences KwaZulu‐Natal Museum Pietermaritzburg South Africa

**Keywords:** Bee pollination, bet hedging, butterfly pollination, exclusion experiment, hawkmoth pollination, mating systems

## Abstract

- The evolutionary limits to generalization in plant pollination systems are often determined by trade‐offs in which adaptations to one set of flower visitors reduces the effectiveness of another set of visitors. A key question is whether flowers can be pollinated equally effectively during the day and the night, given that the attractants for diurnal visitors are expected to be very different to those for nocturnal visitors.
- To address this question, we investigated the pollination system of the mass‐flowering desert geophyte *Nerine laticoma* (Amaryllidaceae) over 2 years. We measured floral traits, including colour, scent, dimensions, floral rewards, visitation and reproductive traits. Finally, we exposed a subset of flowers exclusively to either diurnal or nocturnal visitors to establish their relative contributions to reproduction.
- *Nerine laticoma* has relatively open flowers, with exposed nectar, attracting a wide diversity of pollinators, including bees, butterflies, nocturnal settling moths and hawkmoths. We established that *N. laticoma* is reliant on pollinators for seed production. Flowers exposed only during the day set a similar number of seeds to those exposed only during the night, indicating that the plant is effectively pollinated by both diurnal and nocturnal animals.
- The results highlight the importance of multiple pollinators and their contribution to reproductive success in desert environments with variable pollinator communities. The contribution of all possible pollinators in a system, including frequently overlooked nocturnal visitors, should thus be taken into account.

## Graphical abstract

Both diurnal and nocturnal pollinators are important in the desert plant *Nerine laticoma*.

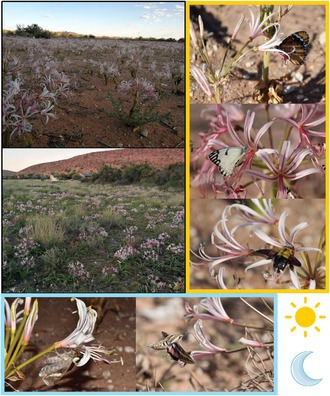

## INTRODUCTION

Pollination systems in plants range from specialized to generalized (Johnson & Steiner 2000). Plants with specialized systems tend to either attract a small subset of potential pollinators via specific advertising signals, restrict access to floral rewards, or use a combination of both sets of filters (Armbruster 2017). Morphological characters, such as narrow corolla tubes and nectar spurs, are often critical for limiting the diversity of animals with access to floral rewards (Stang *et al*. 2006; Botes *et al*. 2008; Rojas‐Nossa *et al*. 2016). Evolutionary specialization, such as developing a deep floral tube and nocturnal anthesis to accommodate one set of visitors, e.g., long‐tongued nocturnal moths, tends to result in trade‐offs that limit the effectiveness of other visitors, e.g., diurnal short‐tongued bees (Phillips *et al*. 2020). Nevertheless, some floral architectures allow plants to utilize a particular primary pollinator, while simultaneously being visited by a whole suite of less efficient pollinations with few trade‐offs (Wenzell *et al*. 2024). One example is flowers with accessible nectar and brush‐like reproductive structures that are often associated with a primary pollinator but also allow many other animals to gain access to rewards and transfer pollen without incurring substantial costs (Amorim *et al*. 2012).

Generalized pollination systems are associated with greater pollinator redundancy as the flowers tend to be more open, with limited restrictions on visitors accessing nectar (Blüthgen & Klein 2011; Armbruster 2017). This can be important in unpredictable habitats such as deserts where flowers tend to be generalized in order to attract as many pollinators as possible during the, usually short, flowering season (Waser *et al*. 1996; Chacoff *et al*. 2012). Generalized plants may exclusively attract either multiple diurnal or nocturnal visitors, or they may attract both diurnal and nocturnal visitors to maximize the opportunity for visitation to flowers. Flowers may do this by secreting nectar continually throughout the flower's life span (e.g., Bertin & Willson 1980; Haber & Franke 1982; Amorim *et al*. 2012), providing a reward for both diurnal and nocturnal visitors. Each flower may also maximize the opportunity for visitation by being long‐lived (Ashman & Schoen 1994; Neiland & Wilcock 1995; Xu & Servedio 2021), and plants may attract more visitors by producing several flowers simultaneously, creating a larger display either at the individual or population level (Thompson 2001). Plants with specialized pollination systems may be at greater risk of not being pollinated than generalized plants, if environmental conditions reduce the availability and abundance of a particular pollinator group (Bond 1994; Dar *et al*. 2006).

When pollinators are unpredictable or scarce, plants are more likely to have some adaptations that enable reproductive assurance (Kalisz & Vogler 2003). The ability to self‐pollinate or reproduce vegetatively is particularly prevalent in plants expanding their ranges or invading new environments (Rodger *et al*. 2013; Pannell *et al*. 2015). The advantage of selfing is most obvious in plants that have become invasive outside of their native range, due to their ability to reproduce in areas where their pollinators are absent (Razanajatovo *et al*. 2016; Le Roux *et al*. 2020). Another possibility is that plants may have high ovule numbers to maximize their reproduction potential in years with optimal pollination conditions (Burd *et al*. 2009).

*Nerine* (Amaryllidaceae, Amaryllidinae) is a relatively small genus of ~25 species endemic to southern Africa (Zonneveld & Duncan 2006; Duncan 2016; POWO 2025). *Nerine* species are widely appreciated as ornamental flowers and numerous hybrids have been developed (Duncan 2014). The pollination of only a handful of these species has been studied in detail and this has revealed the existence of specialized pollination systems involving butterflies (Johnson & Bond 1994), long‐tongued flies (Newman *et al*. 2015, pers obs Theron) and bees (Herman 2012; Newman *et al*. 2015). Specialization in some species such as *Nerine sarniensis* (L.) Herb. which has red flowers that only attract butterflies, seems to be a result of particular advertising traits or morphological fit, rather than morphological filters, because the flowers are relatively open with exposed nectar (Johnson & Bond 1994). *Nerine humilis* (Jacq.) Herb., for example, has open flowers with no restriction to the nectar resources but has long stamens and a stigma that matches the dimensions of the specialized pollinator, the long‐tongued fly, *Prosoeca longipennis* Loew (1858) (Newman *et al*. 2015).

In this study we investigated the pollination biology and breeding system of *Nerine laticoma* (Ker Gawl.) T. Durand and Schinz, a mass‐flowering desert geophyte. We examined the diversity of pollinators to establish the degree of pollination specialization, and the breeding system to determine the reliance of *N. laticoma* on these pollinators. Additionally, we evaluated the individual contribution of diurnal and nocturnal flower visitors to female fitness.

## METHODS

### Study species

This study focused on *Nerine* *laticoma*, one of five summer‐growing *Nerine* species (Duncan 2005, 2016). This species is widely distributed across the dry inland parts of Angola, Zimbabwe, Namibia, Botswana and South Africa (Fig. S1; Harris 2003; Zimudzi *et al*. 2006; Duncan 2016; Duncan *et al*. 2020). It is a variable but distinctive species, comprising two subspecific taxa that differ primarily in the colour and length of their tepals, presence of basal fleshy scales on their filaments, and geographic distribution (Duncan *et al*. 2020). *Nerine laticoma* subsp. *huttoniae* (Schönland) Traub has flowers with deep reddish‐pink or reddish‐maroon tepals, 30–40 mm long, filaments with basal fleshy scales, and is endemic to the Eastern Cape, South Africa. The subspecies *N. laticoma* subsp. *laticoma* differs in having flowers with white or light pink tepals, 30–65 mm long, filaments lacking basal fleshy scales, and is widespread in South Africa and also in the above‐mentioned countries. Both subspecies exhibit variable tepals arrangement, i.e., individuals either with four ascending tepals and two descending tepals, or with five ascending tepals and one descending tepal, can be found within a single population. Here, we focused on subspecies *laticoma*, henceforth referred to as *Nerine laticoma*.

### Study site

This study was conducted on Tswalu Kalahari Reserve, a private game reserve situated in the southern Kalahari Desert within the Northern Cape province of South Africa (27°14′35″S 22°24′18″E; Fig. S1). The same population was sampled across 2 years, from 30 January 2020 to 2 February 2020, and from 26 January 2022 to 6 February 2022. In both years, the population occupied approximately 2 ha at a density of roughly 10 individuals per square meter (Fig. S1). A voucher specimen (M. Castañeda‐Zárate–1692) was prepared and deposited at the Bews Herbarium (NU) of the University of KwaZulu‐Natal.

### Flower anthesis and morphology

To describe the flowering development of *N. laticoma* at our site, we photographed individual flowers daily, from anthesis until flowers withered. We also determined stigma receptivity by hand pollinating flowers on each day after opening. We hand‐pollinated one flower per individual. Furthermore, to obtain a sense of the fit, i.e., contact between anthers and stigmas of *N. laticoma* and its flower visitors, we measured the longest ascending tepal length and functional stigma length using a digital calliper. Tepal length was measured by straightening the longest, middle tepal and measured from the nectary to the tip of the tepal as a proxy for flower size. Functional stigma length was measured on the third day after flower opening. It was measured from the nectary to the stigmatic surface, in a straight line without manipulating the structures, as a measure of the minimum functional pollinator size. To determine if tepal length was associated with functional stigma length, we used a Pearson correlation. All statistical analyses were performed in R (R Core Team 2024).

### Reproductive system and pollen limitation

To assess the dependency of *N. laticoma* on pollinators and degree of pollination limitation at our site, we determined the capacity for autogamous seed set, the degree of self‐compatibility and the effects of supplemental hand‐pollination on seed set. Buds were bagged until they had opened and flowers were at the correct stage for manipulation. Only one flower per treatment was used on each experimental plant, with the total number of treatments per plant depending on the number of flowers available in the appropriate phase (Table 1). Bags were briefly removed to manipulate flowers for the various treatments, after which the bags were replaced until flowers had set seed. To assess self‐compatibility, pollen from flowers within the same plant was used in hand‐pollinations. Unmanipulated buds with exclusion bags were left undisturbed to assess autogamy. Unmanipulated buds without exclusion bags were marked and left undisturbed to assess natural seed set. To assess maximum reproductive output, flowers received supplementary pollen from two pollen donors, at least 2 m away, by removing anthers from donor flowers and rubbing them on all stigma lobes of recipient flowers. Only receptive flowers in the female phase were selected for pollen supplementation. Pollen limitation was calculated as 1 – (unmanipulated/pollen‐supplemented), where 0 indicates no pollen limitation, and 1 indicates complete pollen limitation (Larson & Barrett 2000).

**Table 1:** Mean ± SE number of seeds produced by a single flower per plant after different experimental treatments.

	mean number of seeds (sample size)	
	2020	2022
Natural seed set	4.08 ± 0.52 (39)	3.8 ± 0.34 (65)
Supplemented	5.86 ± 0.71 (21)	3.95 ± 0.52 (20)
Autogamous	0.13 ± 0.09 (24)	‐
Self‐pollinated	0.57 ± 0.23 (23)	‐
Day/night experiment	0.34 ± 0.10 (86)	1.4 ± 0.29 (78)

Day/night experiment refers to the mean of all seed set from the exclusion of pollinators to flowers using mesh bags either at night or day time. See Fig. 6 for a detailed breakdown of the day versus night experiment.

To further describe the sexual system, we determined the pollen and ovule ratio, by counting the number of ovules and pollen grains on unvisited and freshly opened flowers. Buds were collected from the field and allowed to open in the laboratory. The ovaries of 25 flowers were then dissected and the number of ovules present counted under a dissecting microscope. To estimate the total number of pollen grains present in each flower, we collected all of the stamens of 14 flowers and stored them in 1 mL 70% ethanol in Eppendorf tubes. Later, we vortexed the tubes, and added a drop of glycerine to keep the pollen grains in suspension (Kearns & Inouye 1993). We took three samples of 10 μL from each sample and staged the suspension on a microscope slide for counting. The number of pollen grains in each sample was then multiplied by the dilution factor (volume present in the Eppendorf tube/volume withdrawn = 1 mL/0.01 mL). The volume present in the Eppendorf tube was corrected after each sample withdrawal. Values for each sample were averaged to obtain the final estimate of pollen grains per flower.

### Spectral reflectance and scent

To quantify the colour reflectance of *N. laticoma* flowers, we measured the spectral reflectance between the 300–700 nm using an Ocean Optics USB2000 spectrometer (Ocean Optics, Dunedin, FL, USA) from two areas of the middle ascending tepal, on the apex (outer edge) and on the median line. A single tepal from 10 separate individuals was used for measurements. An average spectrum was generated for each of the two tepal areas using the aggplot function in the pavo R package (Maia *et al*. 2019).

To determine if *N. laticoma* flowers, which are unscented to the human nose, produce scent compounds that may attract pollinators, we characterized the chemical composition and emission rates of the flowers. We selected five blooming individuals with flowers in different floral stages. Floral volatiles were collected for 1 h by enclosing each inflorescence in a polyacetate bag (Nalophan^®^, Kalle, Germany) and pumping air from the bag through thermodesorption cartridges filled with 1.5 mg Tenax^®^ and 1.5 mg of Carbotrap^®^ (both Supelco, Bellefonte, PA, USA) at a flow rate of 50 mL min^−1^. The same individuals were sampled during the day (~07:00 h) and at night (~19:00 h). Additionally, a control sample from an empty bag was collected simultaneously using the same method. After sampling, scent traps were sealed in 1 ml glass vials and frozen until the chemical analysis.

We used gas chromatography/mass spectrometry (GC/MS) to analyse the scent samples. The instruments were a Scion 436‐GC with an SGE SolGel Wax standard polar capillary column (30 m × 0.25 mm ID, film thickness: 0.25 μm) coupled to a Scion SQ single quadrupole MS operated in electron‐impact ionization mode at 70 eV. The samples were thermally desorbed using a ChromatoProbe thermal desorption device (Amirav & Dagan, 1997) in a Scion 1079 PTV injector port. The carrier gas was helium, and the column flow rate was 1 ml min^−1^. The injector was held at 40°C for 2 min with a 20:1 split, then increased to 200°C at 200°C min^−1^ in splitless mode. After a 3 min hold at 40°C, the temperature of the GC oven was increased to 240°C at 10°C min^−1^ and held for 12 min. Analysis was carried out using MS Workstation Software v. 8.0.1 for Scion and the NIST17 mass spectral library. Injections of linear alkanes were used to calculate Kovats retention indices (KRI), and identifications were based on matches of KRI and mass spectra patterns. Compounds present in controls were excluded from analysis. Injection of a known amount of octanol was used to calculate emission rates and was injected under identical conditions to all the samples.

### Flower visitors

Overall, we observed flower visitors for 24.5 h over 14 days and 19.3 h over 13 nights (i.e., a total of 43.8 h). In 2020, plants were observed for 8.2 h over 5 days and 6 h over 4 nights, and in 2022 observations were carried out for 16.3 h over 9 days and 13.3 h over 9 nights. We noted the identity of each individual visitor, number of flowers visited within our observation plot, and whether it made contact with the reproductive structures. For statistical analyses, only legitimate visits were used. Additionally, five motion‐triggered cameras (Bushnell NatureView HD Cam Model: 119740, USA) were set up in our plots to document flower visitors during both day and night over 4 days in 2020. Either photographs or videos from a distance of 250 mm or 460 mm were obtained. These were used solely to confirm that no flower visitors were missed during visual observations, as well as for confirmation of visitor frequency and identification.

We used a generalized linear model (GLM) with a negative binomial distribution to analyse whether visitation rate differed between time of day (day or night) as well as among years. As response variable, we used the number of legitimate visits observed on all flowers, with Ln (number of observed flowers) and Ln (observation period in minutes) as offsets. We included year and time of day (day or night) and their interaction as explanatory variable in the model. In addition, we analysed whether visitation rate of each pollinator group differed, using a GLM with a negative binomial distribution. As response variable, we used the number of legitimate visits observed on all flowers in 2020, with Ln (number of observed flowers) and Ln (observation period in minutes) as offset. We included pollinator group as an explanatory variable in the model. For both analyses, Tukey's post hoc tests were performed. All generalized linear mixed model (GLMM)s and GLMs were run using the packages lme4 (Bates *et al*. 2015) and lmerTest (Kuznetsova *et al*. 2017). Marginal means and standard errors were back‐transformed from the scale used in the link function, resulting in asymmetrical error bars, using the package emmeans (Lenth 2024). All Tukey's post hoc tests were run using the multcomp package (Hothorn *et al*. 2008).

Insect flower visitors were collected for identification. In addition, we collected ~ six individuals of each pollinator group, except for nocturnal settling moths, to assess the number of pollen grains deposited as well as the distribution of pollen on the pollinator. Butterflies were collected with nets and immobilized as soon as possible to avoid wing flapping and then euthanised. Hawkmoths and carpenter bees were euthanised with ethyl acetate. Insects were stored individually in plastic bags in the freezer until they could be swabbed for pollen and identified. Pollinators were swabbed multiple times with fuchsin gel across different sections of their bodies. Pollen remaining in the plastic bag was also collected with the fuchsin gel and counted separately. Microscope slides were prepared and pollen counted under a light microscope. We analysed whether the number of pollen grains deposited on the body of the insects differed among insect groups using a GLM with a negative binomial distribution, including number of pollen grains as the response variable and insect group as explanatory variable. Marginal means and standard errors were back‐transformed from the scale used in the link function, resulting in asymmetric error bars. A Turkey's post hoc test was performed to determine if the number of pollen grains differed significantly between pollinator groups.

### Floral rewards

To assess nectar availability and its concentration, we measured standing crop from unbagged individual flowers of different ages, both in the morning and the evening. Either a 5 μl capillary tube (ringcaps^®^ 426; Hirschmann Laborgeräte, Eberstadt, Germany) or a 1 μl capillary tube (1 μl minicap 32 mm end to end; Hirschmann Laborgeräte) was used to extract nectar and measure the volume. Nectar concentration was measured using a Bellingham and Stanley (either 0%–50% or 45%–80%) handheld refractometer. In 2020, nectar volume was measured for 118 flowers (68 flowers during the morning and 50 flowers during the evening) over 3 days. In 2022, nectar volume was measured for 137 flowers (88 flowers during the morning and 49 flowers during the evening) over 4 days. Measurements were performed in the shade to minimize evaporation when placing nectar on the refractometer. We used a GLMM with a negative binomial distribution and Tukey post hoc test to analyse whether nectar volume differed between sunrise and sunset, as well as between years. We included nectar volume as response variable, time of the day and year as (fixed) explanatory variables and flower individual as random factor, as sometimes nectar was measured on multiple days from the same flower. In addition, we analysed whether nectar concentration differed between sunrise and sunset, using a GLM with a negative binomial distribution, including nectar concentration measured in 2022 as response variable, time of the day as (fixed) explanatory variable, and flower individual as random factor. Marginal means and standard errors were back‐transformed from the scale used in the link function, resulting in asymmetric error bars.

### Exclusion experiment

In 2020, we bagged a median of three flower buds per plant and assigned them to either a day (n = 17 plants and 36 flowers) or a night (n = 19 plants and 50 flowers) treatment. All treatment flowers were emasculated in bud. The plants assigned to the day treatment had the experimental flowers unbagged each day at ~06:00–19:00 h for 4 consecutive days after opening of the flower buds. Flowers in the night treatment were unbagged from ~19:00–06:00 h for 4 consecutive nights after the opening of the flower buds. Flowers were then cut just above the bulb and transported back to the lab, where they were allowed to set seed. In 2022 this experiment was repeated with several differences. We bagged one bud or newly opened flower per plant and assigned them to either a day (n = 27) or night (n = 51) treatment. The plants assigned to the day treatment had the experimental flower unbagged for ~13 h (1 day) between sunrise and sunset (06:00–19:00 h) of the female phase of the flower (third day after opening), while flowers of night plants were unbagged for ~11 h (one night) during the night of the female phase of the flower. Flowers were only bagged and unbagged once in order to minimize damage to flowers caused by handling. Flowers were emasculated in bud or before any anthers had dehisced. We used a GLMM with a negative binomial distribution to analyse whether seed set differed between the day and night treatment. We included either the seed set measured in 2020 or 2022 as response variable and treatment and year and their interaction as explanatory variable. Marginal means and standard errors were back‐transformed from the scale used in the link function, resulting in asymmetric error bars.

We also collected the stigmas of the day and night exclusion experiments of 2020, prepared microscope slides with fuchsin gel, and counted the pollen grains under a microscope. We also did this for the open and pollen supplementation treatments. We ran a GLMM with a negative binomial distribution. The response variable was the number of pollen grains, and treatment was the fixed/explanatory variable. Individual was included as random factor as some treatments included more than one flower per plant individual. Treatment included four levels (day, night, natural and pollen supplementation). A post hoc Tukey test was run to determine if the number of pollen grains differed significantly between treatments.

## RESULTS

### Flower anthesis and morphology

Flowers are protandrous, and flower anthesis generally lasts 5 days (Figure S2). The petals of new flowers tend to open early in the morning, followed by dehiscence of two or three out of six anthers a few hours later. The remaining anthers dehisce in the following morning. On the third day, pollen is generally depleted, and anthers begin to wilt. Stigmas then become visibly more swollen and the style starts to curl upwards. From pollination experiments conducted at different stages, it appears that stigmas are most receptive from the third day after opening, with little to no seed produced before this time. Five flowers produced two seeds from supplemental pollen on the first day after opening, and four flowers produced four seeds on the second day after opening. The third and fourth days appeared to be the most fertile, with all the flowers producing some seed (day 3: mean = 5, n = 3; day 4: mean = 1.6, n = 4).

Size of flowers was highly variable across individual plants (mean tepal length = 42.2 mm ± SE = 1.8; range = 28–55 mm), as was stigma length (mean = 38.4 mm ± SE = 0.5; range = 18–58 mm) (Figure S3). Tepal length was positively associated with the functional stigma length (r = 0.805, *P* = 0.001; Figure S4).

### Reproductive system and pollen‐limitation

*Nerine laticoma* appears to set little to no seeds autogamously (2020: mean = 0.13; range = 0–2) and, likewise, pollen from within the same plant produced low seed set (Table 1). Natural seed set was quite variable between flowers (combined 2020 and 2022 mean: = 3.9 seeds per flower; range = 0–12; Table 1), and seed production was slightly pollen‐limited (2020: 0.32; 2022: 0.03). Flowers produced substantially more pollen than ovules (pollen: 87828 ± 3506; ovule: 5 ± 0.65) with a pollen/ovule ratio of 17565.

### Spectral reflectance and scent

Flower colour was variable across individuals, ranging from white to bright pink. Reflectance spectra of both the apex and median line of the tepals showed an increase from 350 to 440 nm. Most flowers were a light pink colour with a darker pink median line (Figure S5).

Flowers of *N. laticoma* were unscented to the human nose and emitted very few volatile compounds. Only seven compounds (all aliphatics) were recorded, and these comprised unsaturated aldehydes and alcohols, as well as saturated alcohols (Table 2). The total emission rate (in ng per flower per hour) did not differ between day and night samples (7.37 ± 2.35 versus 5.19 ± 1.63, *t* = 0.74, *P* = 0.5, paired *t*‐test).

**Table 2:** Volatile compounds recorded in the scent emitted by flowers of *Nerine laticoma*.

compound	RT (mins)	estimated KRI	published KRI	night samples (n = 5)	day samples (n = 5)
(E)‐2‐Octenal	11.87	1419	1429	7.73 ± 1.15 (5)	11.08 ± 1.95 (5)
1‐Octen‐3‐ol	12.02	1432	1450	14.85 ± 8.35 (5)	5.65 ± 1.35 (5)
Heptanol	12.08	1437	1453	6.22 ± 0.5 (5)	5.23 ± 0.82 (5)
Octanol	13.38	1546	1557	53.86 ± 6.02 (5)	55.49 ± 8.62 (5)
(E)‐2‐Decenal	14.53	1645	1644	2.41 ± 0.64 (5)	4.13 ± 0.67 (5)
Nonanol	14.61	1652	1660	12.9 ± 3.63 (5)	15.14 ± 4.21 (5)
Decanol	15.8	1756	1760	2.02 ± 0.65 (5)	3.28 ± 0.67 (5)
Emission rate (ng flower^−1^ h^−1^)				5.19 ± 1.63	7.37 ± 2.35

KRI = Kovats retention index as estimated from RTs relative to flanking alkanes and as in the NIST 2020 database. Values are mean (± SE) proportions, with the number of samples in which a compound was recorded in parentheses. RT = retention time.

### Flower visitors

**Fig. 1:**
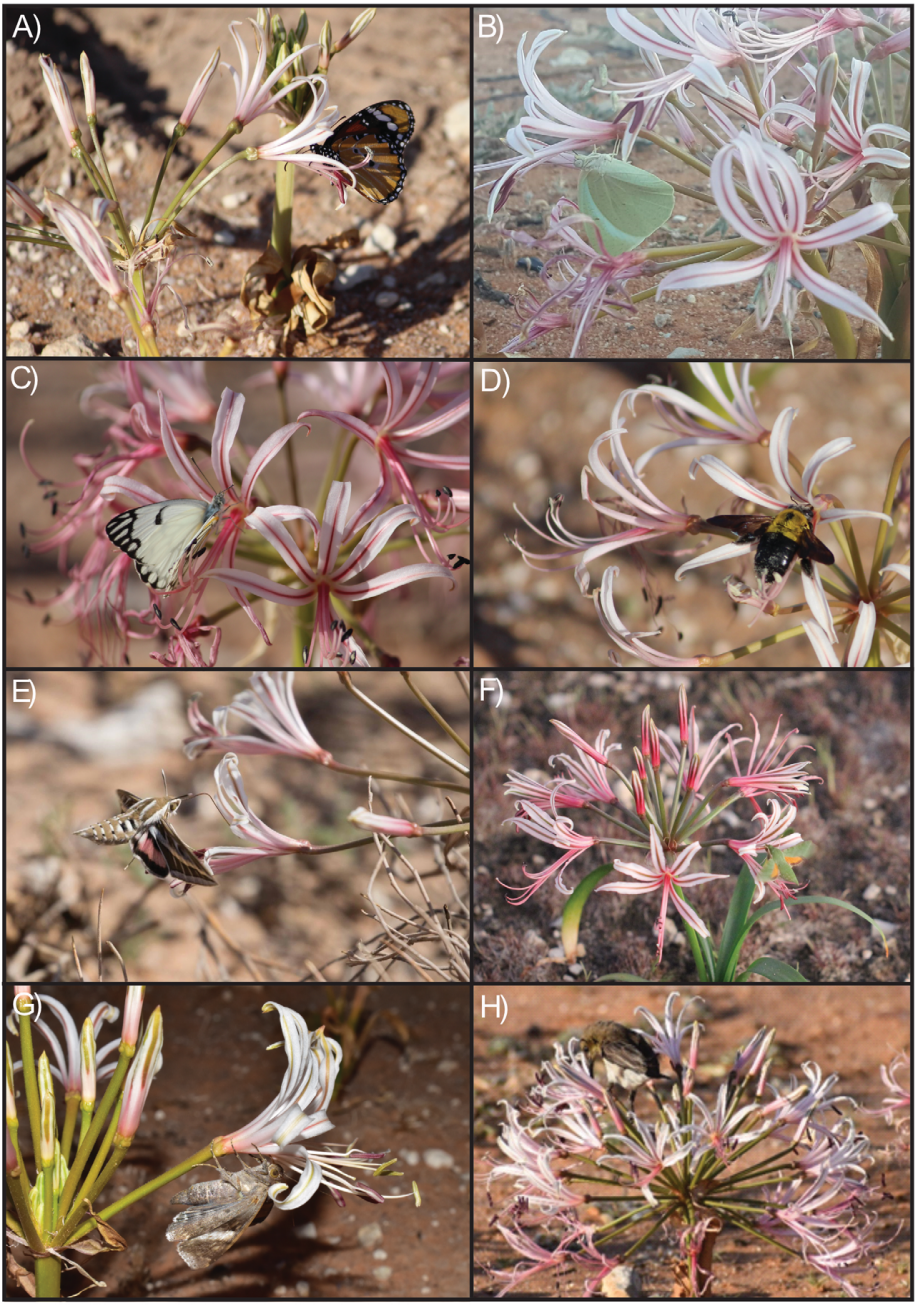
Insect visitors to flowers of *Nerine laticoma*. (A) *Danaus chrysippus* (Monarch butterfly), (B) *Catopsilia florella* (African migrant butterfly), (C) *Belenois aurota* (Pioneer white butterfly), (D) *Xylocopa senior* (Carpenter bee), (E) *Hyles livornica*. (Striped hawkmoth), (F) *Basiothia medea* (Small verdant hawkmoth), (G) Nocturnal settling moth, (H) *Cinnyris fuscus* (Dusky sunbird).

**Fig. 2:**
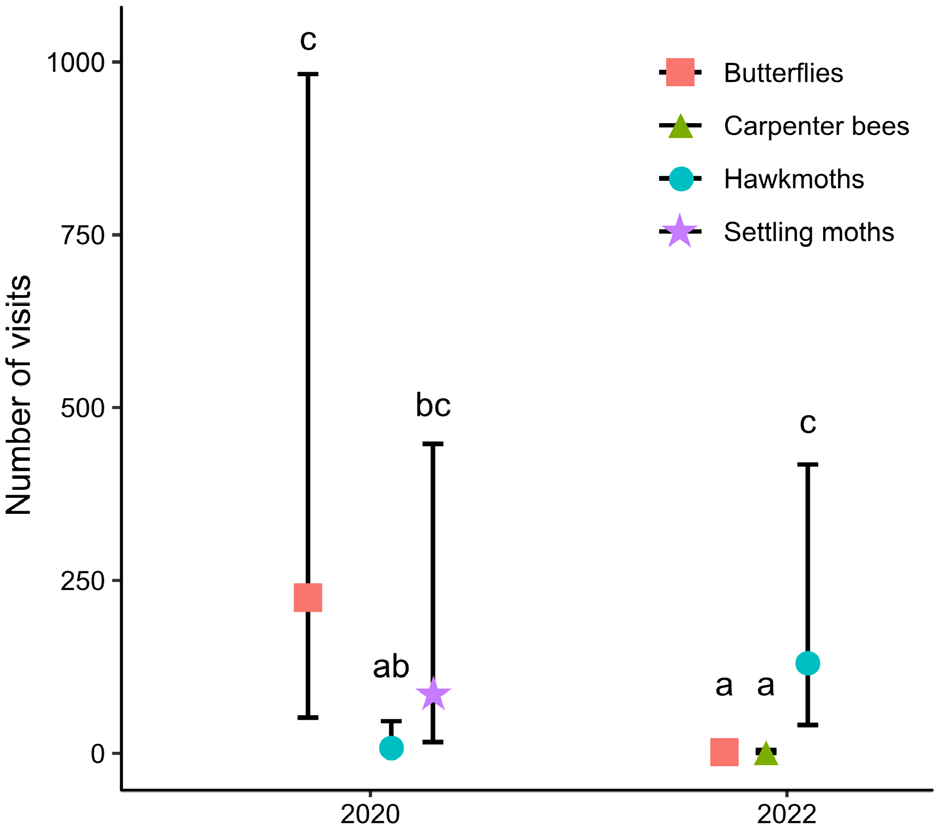
Mean ± SE number of visits to flowers by pollinator groups in 2020 (8.2 h of observations over 5 days, 6 h over 4 nights) and 2022 (16.3 h over 9 days, and 13.3 h over 9 nights). Nocturnal settling moths were absent in 2022. In 2020, most butterfly visits were made by the two migratory species (*Catopsilia florella* and *Belenois aurota*) accounting for 91% of butterfly visits. Pollinator groups sharing the same letter do not differ significantly.

The flowers of *N. laticoma* were visited primarily by butterflies (Nymphalidae and Pieridae), carpenter bees (Apidae: *Xylocopa*), hawkmoths (Sphingidae), and nocturnal settling moths (Fig. 1A–G). Diurnal visitors included three migratory species of butterfly: *Belenois aurota* Fabricius (1793), *Catopsilia florella* Fabricius (1775), and *Danaus chrysippus* Linnaeus (1758); two species of carpenter bee, *Xylocopa caffra* Linnaeus (1767) and *X. senior* Vachal (1899). Nocturnal visitors included at least two species of hawkmoth: *Hippotion celerio* Linnaeus (1758) and *Hyles livornica* Esper (1780), and nocturnal settling moths such as *Cyligramma latona* Cramer (1775) were also commonly observed. Surprisingly, we also recorded one case of visitation by a male dusky sunbird (*Cinnyris fuscus* Vieillot (1819)) extracting nectar from several individuals (Fig. 1H). However, both the sunbird and many of the settling moths were unable to make contact with the anthers as they approached and accessed the nectar from behind the corolla. In 2022, only carpenter bees and hawkmoths were observed visiting regularly, with butterflies virtually absent and settling moths being absent during this period (Fig. 2). During 2022, we recorded three individuals of the hawkmoth *Basiothia medea* Fabricius (1781), a species not seen in 2020.

Of the three butterfly species observed, *D. chrysippus* tended to visit legitimately (Fig. 1A). Their mean (±SE) pollen loads were 444 ± 149 (Fig. 4). The two other migratory butterflies, however, performed poorly as pollinators, with many instances of thieving (Fig. 1B) and low pollen loads when they did make contact (Fig. 1C) (*C. florella*: 39 ± 20; *B. aurota*: 87 ± 27; Fig. 4). Only 30.7% of all visits were considered legitimate, as visitors often failed to contact the reproductive structures. Both *C. florella* and *B. aurota* carried significantly less pollen than carpenter bees (Z = 4.459, *P* < 0.001 for *C. florella*; Z = 3.429, *P* = 0.006 for *B. aurota*), hawkmoths (Z = 3.660, *P* = 0.002; for *C. florella*; Z = 2.537, *P* = 0.083 for *B. aurota*) and *D. chrysippus* (Z = 3.859, *P* = 0.001 for *C. florella*; Z = 2.786, *P* = 0.043 for *B. aurota*). Carpenter bees generally had the highest pollen load (647 ± 268; Fig. 4), concentrated around the dorsal and ventral sides of the abdomen (Fig. 1D). *Danaus chrysippus* had the second highest pollen load, followed by hawkmoths (Fig. 1E, F; 361 ± 188; Fig. 4). However, pollen loads of carpenter bees, *D. chrysippus* and hawkmoths did not differ significantly (Z = 0.602, *P* = 0.975 for carpenter bees – *D. chrysippus*; Z = 0.969, *P* = 0.869 for carpenter bees – hawkmoths; Z = 0.344, *P* = 0.997 for hawkmoths – *D. chrysippus*; Fig. 4).

**Fig. 3:**
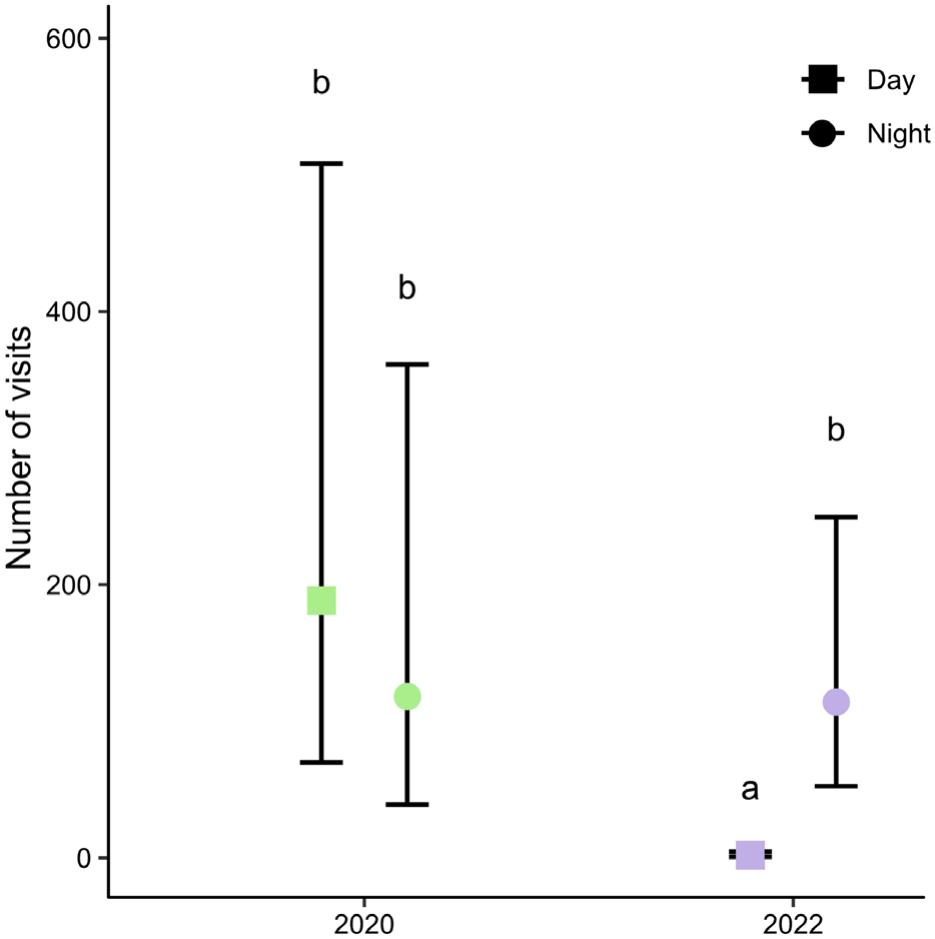
Mean ± SE number of visits (legitimate visits only) to flowers observed during the day and at night in 2020 and 2022. Treatments sharing the same letter do not differ significantly.

Visitation rate and flower visitors and especially their relative abundance differed among 2020 and 2022. In 2020, overall visitation rate was significantly higher compared to 2022 (Z = 8.668, *P* < 0.001). While in 2020, visitation rate did not significantly differ between day and night (Z = 0.772, *P* = 0.867), in 2022 visits were significantly more frequent at night than during the day (Z = 8.723, *P* < 0.001; Fig. 3). In 2020, butterflies were the most frequent day visitor, while settling moths were the most common nocturnal visitor. Visitation rate of butterflies was significantly higher than hawkmoths (Z = 3.821, *P* = 0.002) but was not significantly different from the visitation rate of settling moths (Z = 1.153, *P* = 0.859). Visitation rates of settling moths did not significantly differ from those of hawkmoths (Z = 2.589, *P* = 0.100). In 2022, at night we only observed hawkmoths, and their visitation rate was higher than the visitation rate of both butterflies (Z = 6.913, *P* < 0.001) and carpenter bees (Z = 7.036, *P* < 0.001). Visitation rate of butterflies and carpenter bees did not differ significantly (Z = 0.172, *P* = 1.000).

**Fig. 4:**
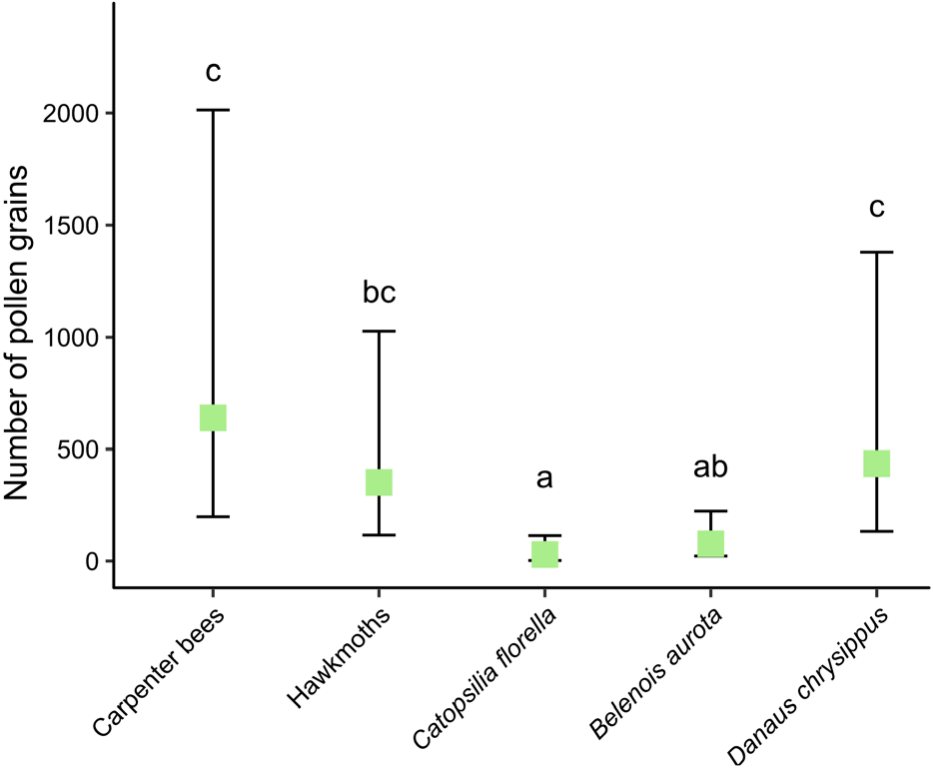
Mean ± SE number of pollen grains counted from the body of each group of pollinator, with butterflies separated into their separate species. Counts were taken from pollinators caught in 2020 (Carpenter bees, *Catopsilia florella*, and *Danaus chrysippus* n = 6, *Belenois aurota* n = 8 and hawkmoths n = 7). Pollinator groups sharing the same letter do not differ significantly.

**Fig. 5:**
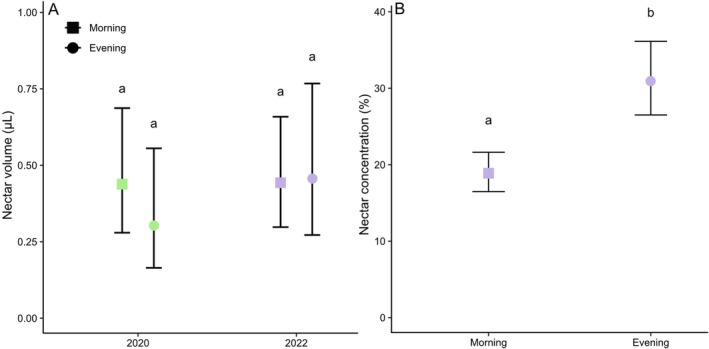
(A) Mean ± SE nectar volume measured just after sunrise (morning) and just before sunset (evening) in 2020 (n = 134) and 2022 (n = 179). (B) Mean ± SE nectar concentration measured in the morning (n = 118) and the evening (n = 61) of 2022. Treatments sharing the same letter do not differ significantly.

**Fig. 6:**
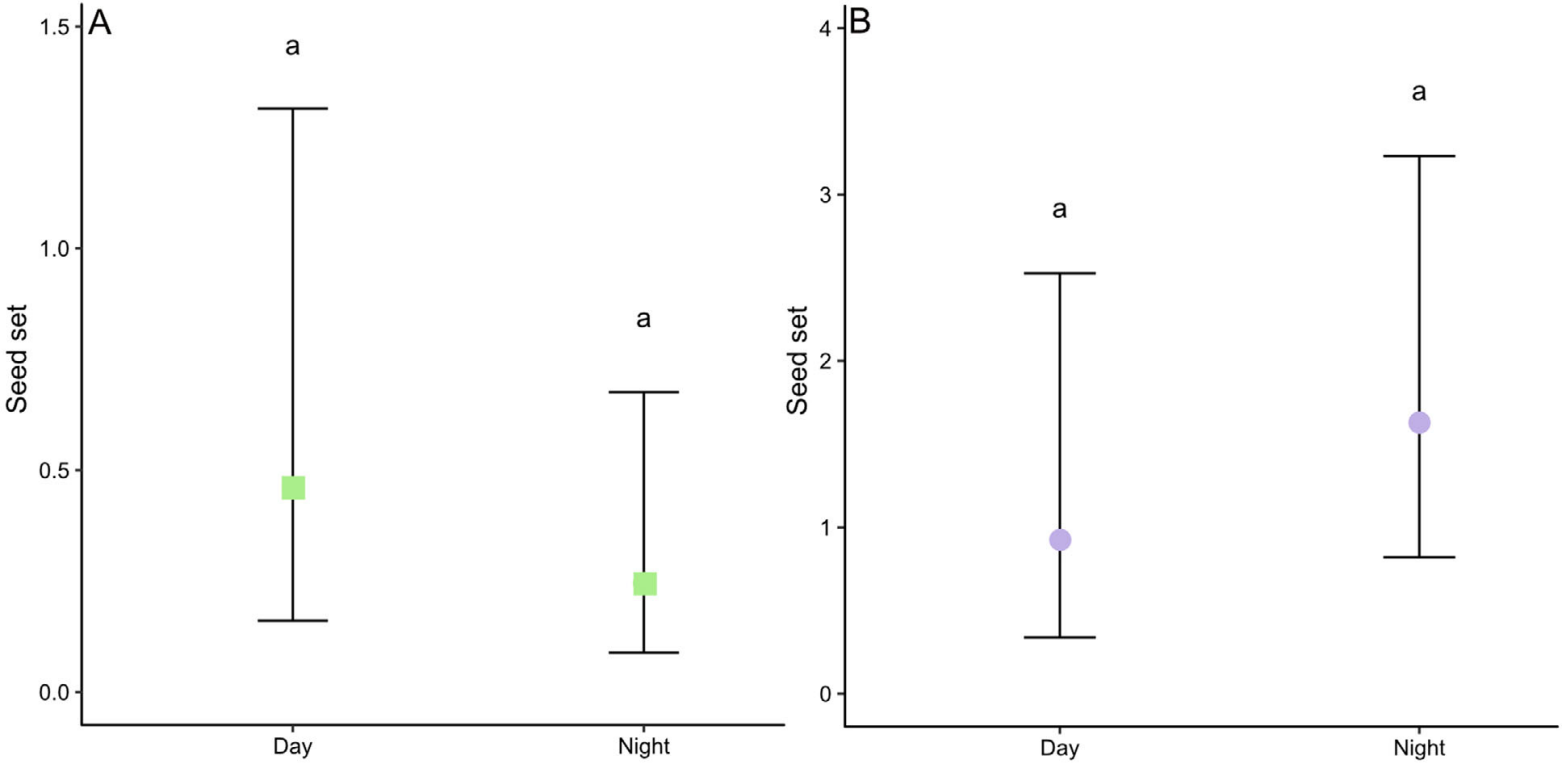
Mean ± SE seed set produced per flower by nocturnal and diurnal exclusion experiments in 2020 (A) and 2022 (B). In 2020, 86 flowers, each on a different plant, were exposed to visitors for either 4 nights or 4 days, while in 2022 78 flowers were exposed to visitors for a single day or for a single night. Treatments sharing the same letter do not differ significantly.

**Fig. 7:**
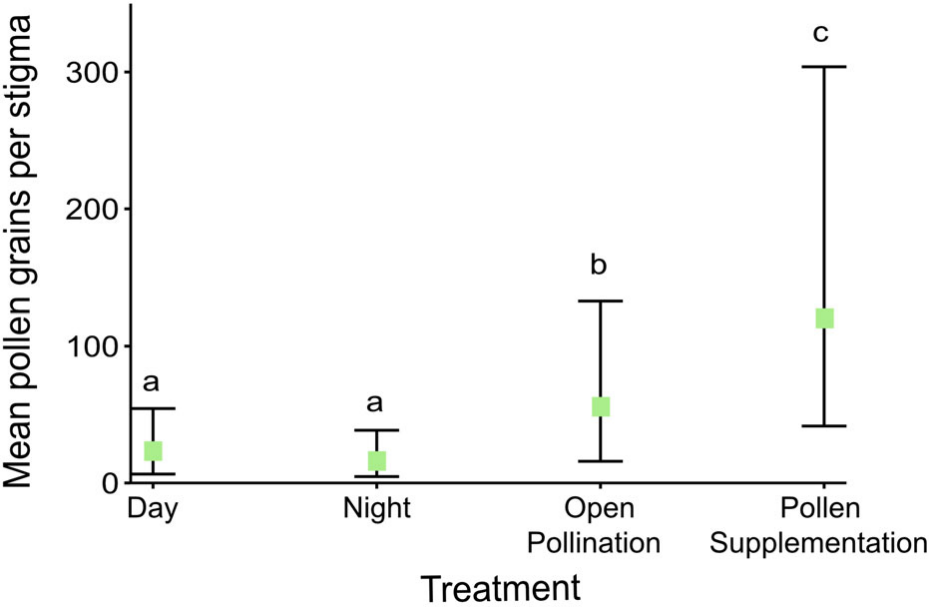
Mean number of pollen grains per stigma from different experimental treatments in 2020 (day n = 17, night n = 19, open n = 29, pollen supplementation n = 19). Day and night were bagged exclusion treatments, where the name indicates the period that visitors were able to access flowers. Values are means ± SE. Treatments sharing the same letter do not differ significantly.

### Flower rewards

The overall nectar volume did not differ among years (Z = 1.311, *P* = 0.190). While nectar volume sampled at sunrise was higher than nectar volume sampled at sunset in 2020, this difference was not significant (Z = 1.241, *P* = 0.601). In 2022, there was no significant difference in nectar volume sampled at sunrise or sunset (Z = 0.119, *P* = 0.999; Fig. 5). Mean (±SE) nectar concentration was significantly higher in the evening (30.9% ± 2.5%) than in the morning (20.3% ± 0.8%; Z = 5.527, *P* < 0.001).

### Exclusion experiment

In 2020, mean (± SE) seed set was slightly higher for flowers that were open during the day (0.48 ± 0.11) than those that were open during the evening (0.47 ± 0.18), however there was no significant difference between the two treatments (Z = 0.963, *P* = 0.336; Fig. 6). The number of pollen grains found on the stigmas was associated with treatment type (day exclusion, night exclusion, open and pollen supplemented; χ^2^_(3)_ = 78.78, *P* < 0.001). The post hoc Tukey test indicated that all treatments were significantly different from each other (*P* < 0.01) except for day and night (*P* = 0.5; Fig. 7).

Seed set collected in 2022 was generally higher for flowers that were open during the evening (1.6 ± 2.74) than those that were open during the day (0.9 ± 2.14), however, there was no significant difference between the two treatments (Z = 1.041, *P* = 0.298; Fig. 6). Flowers which had been exposed to only diurnal or only nocturnal pollinators set fewer seeds (1.4 ± 0.34; Table 1) than those that could be visited by all pollinators (3.8 ± 2.75; Table 1).

## DISCUSSION

The results presented here suggest that *Nerine laticoma* is an obligate outcrosser with a generalized pollination system, in which both diurnal and nocturnal pollinators are effective. Furthermore, *N. laticoma* attracts a number of functionally different pollinators, both within a season and across years. The large floral size variation, colour variation and lack of significant scent found within our study population aligns with a profile for a generalized pollination syndrome. Overall, our findings indicate that the reproductive strategy of *N. laticoma* to distribute risk, may be favoured in arid environments where conditions can be unfavourable for certain pollinators in a given year. This is highlighted by the dramatic difference in pollinator assemblages across the 2 years of the study.

The results of the reproduction experiments suggest that *N. laticoma* is partially self‐compatible but is highly dependent on the diverse array of pollinators to set seeds as it cannot self‐pollinate to any significant degree. This is in accordance with what is known for *N. laticoma* subsp. *huttoniae* (Duncan 2016), and other *Nerine* species, such as *N. humilis* which is also reliant on pollinators for fertilization (Newman *et al*. 2015). There are, however, some reports of *Nerine* species with small flowers, such as *N. ridleyi*, being self‐compatible (Duncan 2008). Although we cannot exclude the effect of handling, seed set was substantially reduced when plants were exposed to either nocturnal or diurnal pollinators only, suggesting that both groups of pollinators are required to maximize female fitness. The reduction in fruit set and seed set per fruit observed in 2022 may have been further exacerbated by the experimental design, as flowers were only exposed to pollinators for ~12 h instead of the full 2–3 days that stigmas are receptive. Nevertheless, the similarity in seed set observed in both years in the day/night exclusion treatments strongly suggest that both groups, diurnal and nocturnal, contribute to similar extents to the reproductive success of *N. laticoma*. In mixed butterfly‐ and hawkmoth‐pollinated systems, such as *Habenaria* Willd. (Orchidaceae), it is not uncommon for both groups to contribute more or less equally to the reproductive success of the species (Tan *et al*. 2023). However, this is not the case for all generalized systems, as is seen in *Silene alba* (Mill.) E.H.L.Krause (Caryophyllaceae), where nocturnal pollinators outperformed diurnal pollinators in both visitation rate and seed set produced (Young 2002).

The mass‐flowering phenomenon, in addition to the open morphology of these flowers, without substantial restrictions to nectar, likely contributes to the diversity of pollinators attracted to the flowers (Martínez‐Harms *et al*. 2022). Carpenter bees, monarch butterflies (*D. chrysippus*) and hawkmoths carried the largest pollen loads, indicating potentially higher pollination efficiency. Of these three most efficient functional pollinator types visiting *N. laticoma*, hawkmoths had the highest visitation rate, which is surprising for a plant with very low levels of emission of volatile compounds. Scent is often a large component of flower attraction in moth pollination systems (Knudsen & Tollsten 1993; Balkenius *et al*. 2006; Powers *et al*. 2020). The floral scent of *N. laticoma* was extremely weak and comprised of aliphatic compounds, which are not characteristics of moth‐pollinated flowers (Farré‐Armengol *et al*. 2020). The emission rate did not vary between day and night periods and was just 5–7 ng flower^−1^ h^−1^, which is orders of magnitude lower than the emissions rates of c. 100–1000 ng flower^−1^ h^−1^ that are typically reported for plants pollinated by moths (Dötterl *et al*. 2012; Balducci *et al*. 2020; Farré‐Armengol *et al*. 2020). Some reported values for moth‐pollinated plants have been even higher, such as 9.1 ng flower^−1^ h^−1^ in *Bonatea steudneri* (Rchb.f.) T.Durand & Schinz (Orchidaceae) (Balducci *et al*. 2019) and 24 ng flower^−1^ h^−1^ in *Chamaepentas nobilis* (S.Moore) Kårehed & B.Bremer (Rubiaceae) (Johnson 2024). However, it is clear that moths do also visit some flowers with very low levels of scent production. Moths were regular visitors to flowers of a South Africa *Erica* L. species (Ericaceae) with nocturnal scent emission of just 26 ng flower^−1^ h^−1^ (van der Niet & Cozien 2024) and very low levels of scent emission (5–26 ng flower^−1^ h^−1^) were also recorded in moth‐pollinated *Schiedea* Cham. & Schltdl. flowers (Caryophyllaceae) (Powers *et al*. 2020). Species with low scent emission rate per flower often have large numbers of very small flowers clustered in each inflorescence. For better comparisons, scent emission data should ideally be standardized by flower dry mass (Majetic *et al*. 2015), but that is seldom recorded in any study, including the current one.

It has been noted in a number of butterfly pollination systems that attraction of the insects to flowers may be based mainly on colour (Newman *et al*. 2012; Chen *et al*. 2021), but there is also some evidence that attraction of some butterflies to flowers can be mediated by scent (Andersson & Dobson 2003; Kiepiel & Johnson 2021). Similarly, carpenter bees visit flowers that are not scented to the human nose, but do also respond to scent compounds (Rabeschini *et al*. 2021).

Nocturnal settling moths were often observed to rob nectar from flowers, as they were usually too small, or in an inappropriate orientation to make contact with reproductive structures (Fig. 1G). Similarly, diurnal *B. aurota* and *C. florella* butterflies also often functioned as nectar robbers, as they frequently visited in a position that did not allow legitimate pollination to occur (Fig. 1B). Similar to other pollination systems, the pollinators that were less efficient pollen carriers were often the most abundant pollinators, when present (e.g., Rivera‐Marchand & Ackerman, 2006). Notably, their presence fluctuated considerably across years. During 2022, settling moths and all three butterfly species were virtually absent compared to our first year of study. Although seed set was lower in 2020, when butterflies and settling moths were present, than in 2022 it is unlikely that there is a large trade‐off involved in having these additional visitors, as they can occur in large numbers. While it cannot be ruled out that these additional, less efficient, pollinators may deter hawkmoths or carpenter bees in some way, they likely provide a degree of reproductive assurance to the plants (Waser *et al*. 1996).

Our results highlight the importance of both diurnal and nocturnal flower visitors present in this system, as they appear to contribute equally to reproductive success. We also demonstrate the importance of examining the contribution of all possible flower visitors in a system. This is especially noteworthy for nocturnal pollinators that are often neglected due to observer bias or logistical difficulties associated with working at night (Macgregor & Scott‐Brown 2020; Alison *et al*. 2022; Fijen *et al*. 2023).

## AUTHOR CONTRIBUTIONS

All authors contributed to conception and design of the study. GLT coordinated all research activity planning and execution. GLT, SGTK, CD, CB, MCZ performed data collection, curation. GLT, SGTK, CD, SDJ permformed the statistical analyses. All authors contributed equally to interpretation of results and writing of the final version.

## Supporting information


**Figure S1.** Site and locality information, with field photographs demonstrating the plant density in a small part of the greater population in (A) 2020 and (B) 2022, as well as a map of the range of *Nerine laticoma* and the site location indicated.

**Figure S2.** Photographs of *Nerine laticoma* flowers in various stages of development. (A) First day of flowering, newly opened, (B) three anthers dehisced, (C) six anthers dehisced, (D) stigma receptive, (E) last day of flowering, stigma receptive.

**Figure S3.** Photograph of the floral size variation in the population of *Nerine laticoma*.

**Figure S4.** Correlation between functional stigma length on the third day after flower opening and the middle tepal length.

**Figure S5.** Spectral reflectance of two areas on the longest ascending tepal of *Nerine laticoma* flowers, (A) apex, (B) median line.
