# Supplementary figures and images for: Generalization for both diurnal and nocturnal pollination in the mass‐flowering desert geophyte Nerine laticoma (Amaryllidaceae)
Source: Plant Biol (Stuttg). 2025 Dec 12;28(2):468–78. doi: 10.1111/plb.70153 (PMC12884022; doi:10.1111/plb.70153)

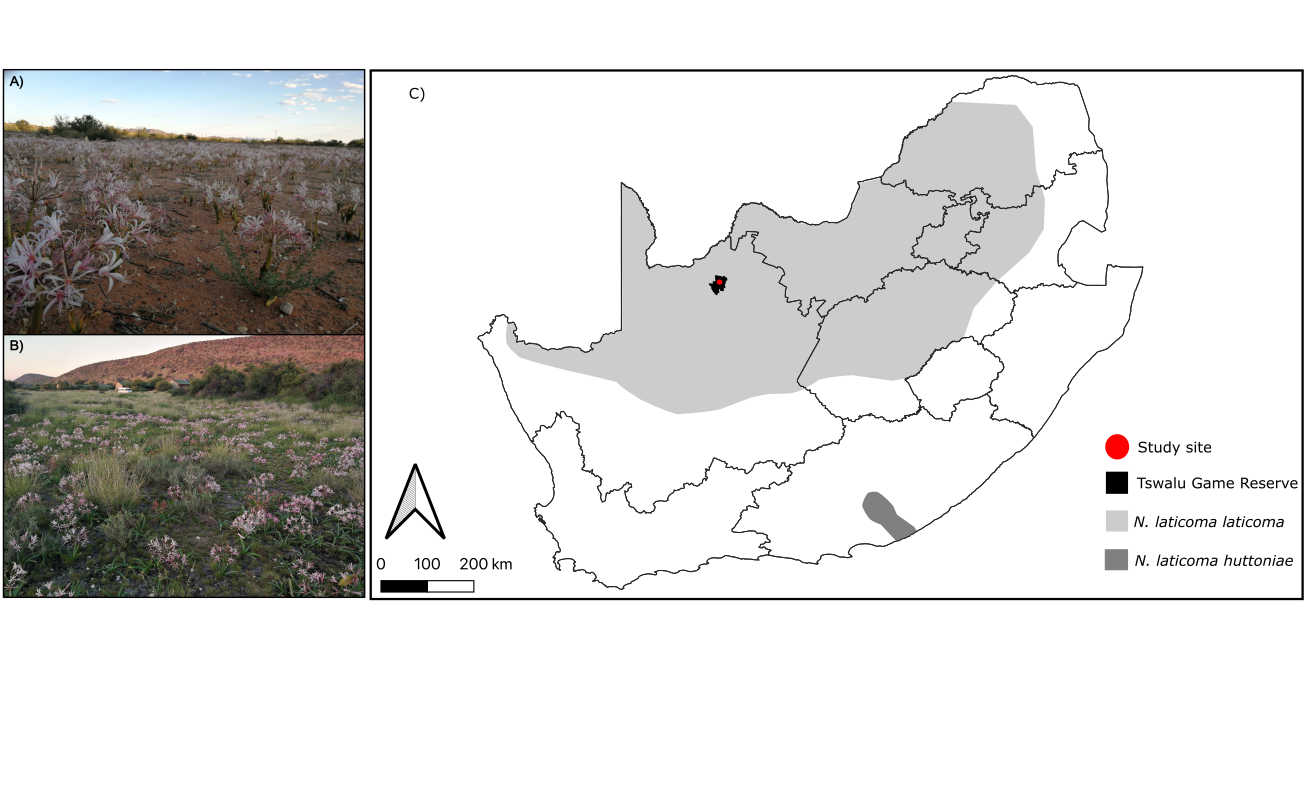

Supplement: Supplementary file 1 — Figure S1. Site and locality information, with field photographs demonstrating the plant density in a small part of the greater population in (A) 2020 and (B) 2022, as well as a map of the range of Nerine laticoma and the site location indicated. [file PLB-28-468-s005.png]

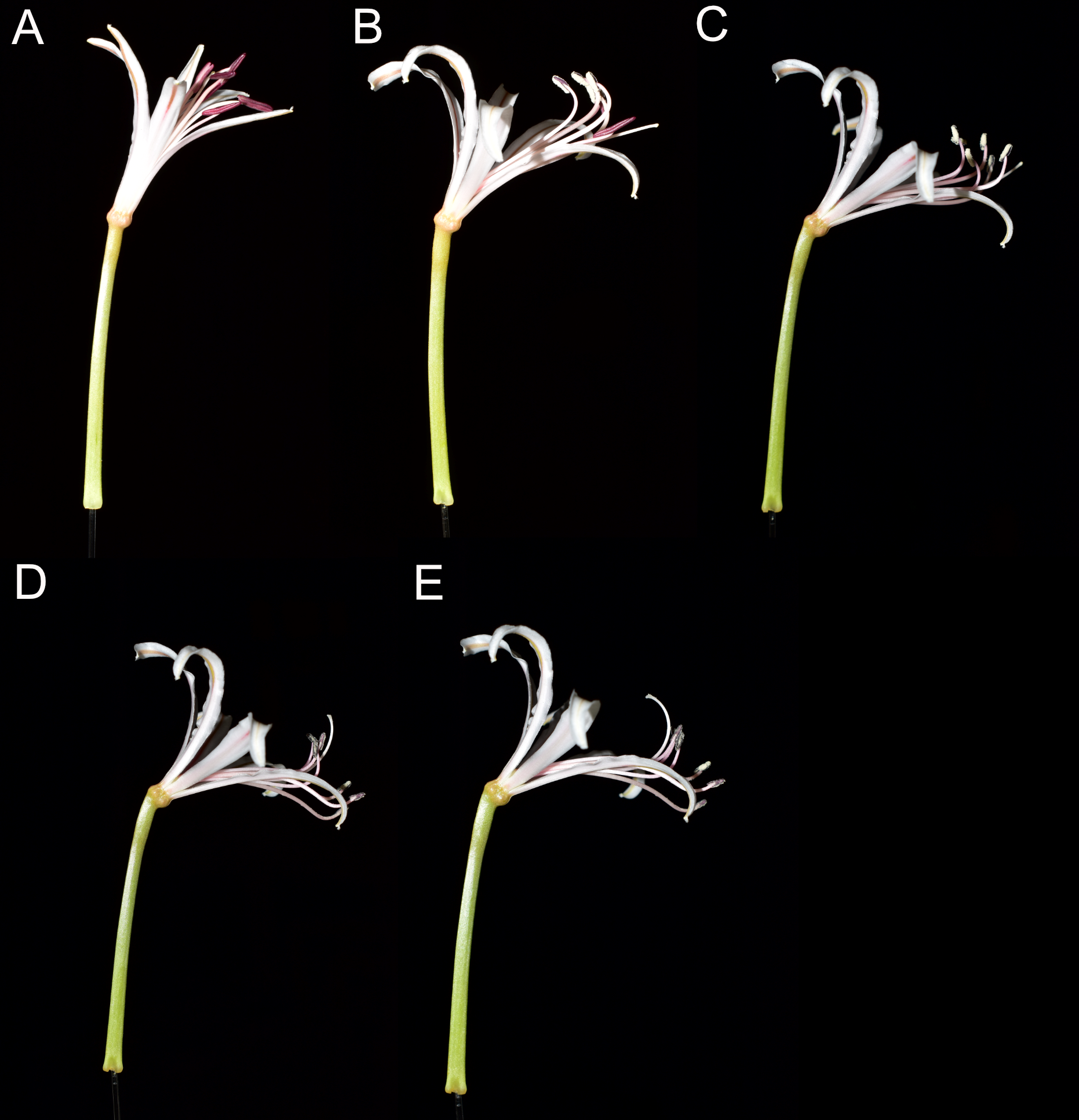

Supplement: Supplementary file 2 — Figure S2. Photographs of Nerine laticoma flowers in various stages of development. (A) First day of flowering, newly opened, (B) three anthers dehisced, (C) six anthers dehisced, (D) stigma receptive, (E) last day of flowering, stigma receptive. [file PLB-28-468-s004.png]

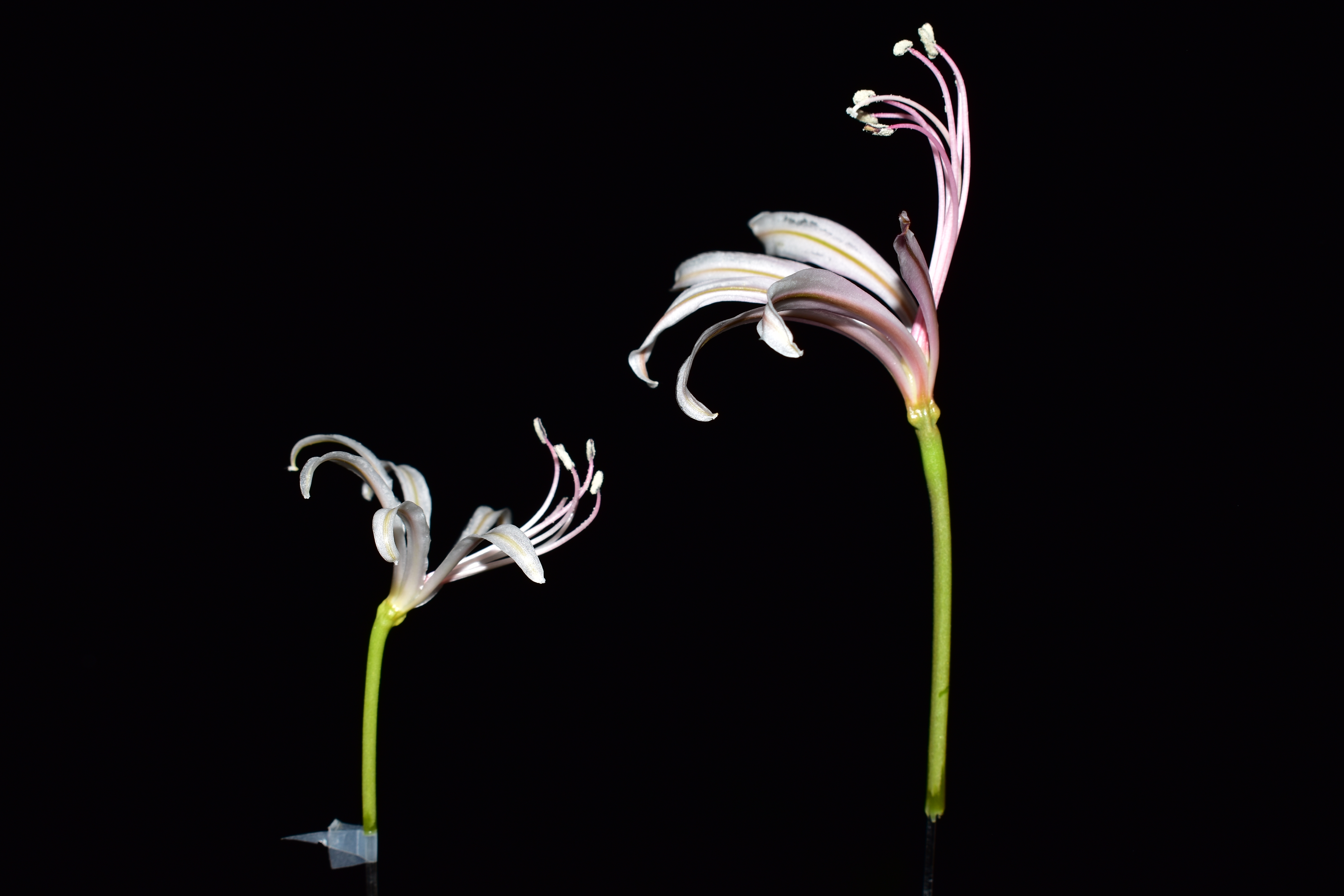

Supplement: Supplementary file 3 — Figure S3. Photograph of the floral size variation in the population of Nerine laticoma. [file PLB-28-468-s003.jpg]

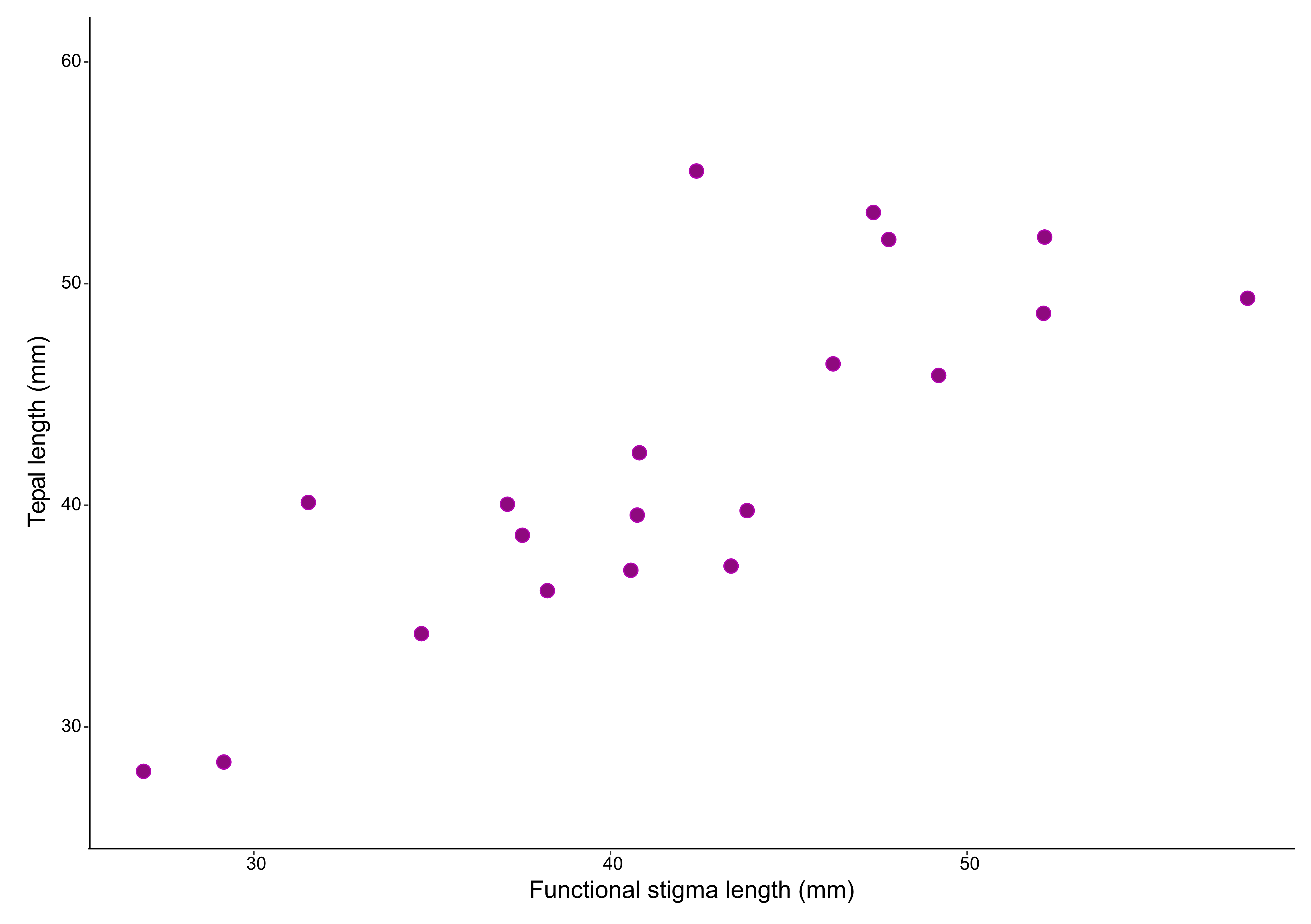

Supplement: Supplementary file 4 — Figure S4. Correlation between functional stigma length on the third day after flower opening and the middle tepal length. [file PLB-28-468-s002.png]

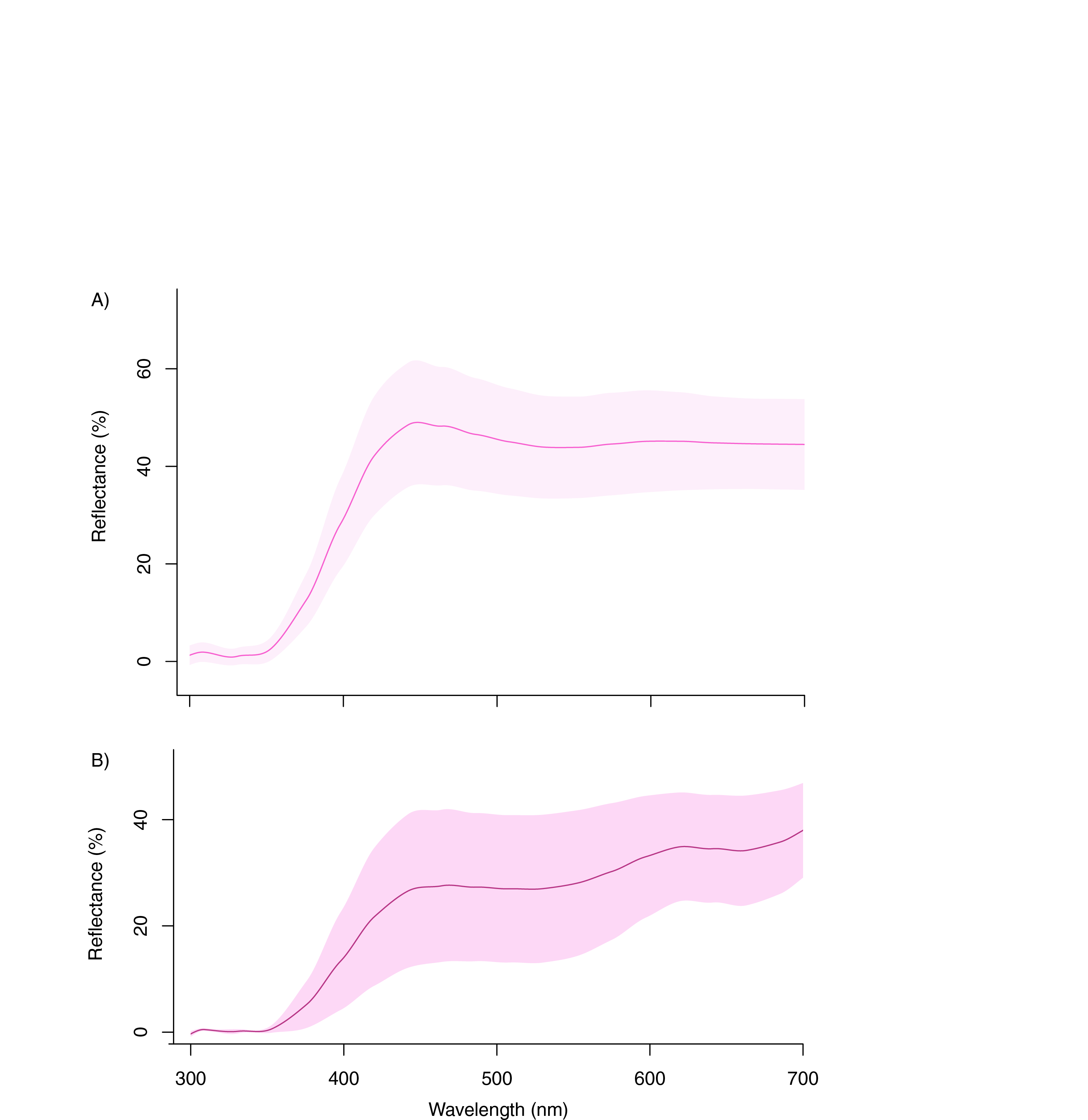

Supplement: Supplementary file 5 — Figure S5. Spectral reflectance of two areas on the longest ascending tepal of Nerine laticoma flowers, (A) apex, (B) median line. [file PLB-28-468-s001.png]
